# Supplementary material for: Decisions and choices about fertility and family planning: Perspectives from husbands and wives in Sudan
Source: PLoS One. 2026 Mar 13;21(3):e0343941. doi: 10.1371/journal.pone.0343941 (PMC12987423; doi:10.1371/journal.pone.0343941)
Supplement: S1 Appendix — (DOCX) [file pone.0343941.s001.docx]

S1 Appendix:

Interview guide for the husband participants.

Section 1: Please provide information about employment, education, age, socio-economic status, religion, and the number of your children.

Section 2:

Can you please share your upbringing and how it impacts your decisions and position in your household?

What is your knowledge about family planning and its methods (modern/natural).

What are your perceptions of family planning use (good, beneficial, harmful, bad, don’t know, etc.)

Do you think it is important for you or your wife to use family planning and why?

What are your perceptions about fertility

Do you think it is important to have children, and why?

Who do you think should be the primary decision-maker in family planning use, the wife or the husband, and why?

Do you have discussions with your wife about family planning use/need?

Do you support/oppose your wife’s contraceptive use?

Do you accompany your wife to the family planning center?

What factors might influence your perceptions about your wife’s family planning use (wife’s medical reasons, wife’s contraceptive need, fertility/family size, masculinity/virility, head of household, traditions/norms, religious reasons).

Interview guide for the wife participants.

What is your knowledge about family planning and its methods (modern/natural).

Do you think it is important to use family planning, and why?

What are your perceptions of family planning use (good, beneficial, harmful, bad, don’t know, etc).

What are your perceptions about childbearing?

Do you think it is important to have children, and why?

Are you currently using contraceptives or used them in the past, which type?

Where do you obtain your contraceptives from?

Have you ever used contraceptives covertly? And why.

Who do you discuss your family planning use/need with (husband, family planning provider, etc).

Do you think your husband knows about contraceptives.

Do you think it's important for your husband to know or be involved in family planning?

Do you think your husband supports/opposes your contraceptive use.

Does your husband ever accompany you to the family planning center.

Who do you think is the primary decision-maker in your family planning use (wife or husband) and why.

What factors might influence your opinions about your own family planning use (medical reasons/doctor's advice, contraceptive need, fertility/childbearing norms, socio-cultural reasons, husband influence, woman’s choice/decision/right, etc.).
