# Supplementary material for: Decisions and choices about fertility and family planning: Perspectives from husbands and wives in Sudan
Source: PLoS One. 2026 Mar 13;21(3):e0343941. doi: 10.1371/journal.pone.0343941 (PMC12987423; doi:10.1371/journal.pone.0343941)
Supplement: Attachment — Submitted filename: Response to Reviewers.docx [file pone.0343941.s002.docx]

Corresponding author: Dina Badri,
Ahfad University for Women, Sudan.

email: badrid21@gmail.com

Co-author: Professor Anja Krumeich, Dept. of Health, Ethics and Society, Maastricht University, CAPHRI,

Universiteitssingel 40, 6229 ER, Maastricht,

The Netherlands.

Email: [a.krumeich@maastrichtuniversity.nl](mailto:a.krumeich@maastrichtuniversity.nl)

Co-author: Professor Bart Van de Borne, Dept. Of Health Promotion, Maastricht University, CAPHRI, Universiteitssingel 40, 6229 ER, Maastricht, The Netherlands.

Email: [b.vdborne@maastrichtuniversity.nl](mailto:b.vdborne@maastrichtuniversity.nl)

Date: 20 December 2024

To the Academic Editor PLoS One

Subject: Re-submitting the revised manuscript. Title: Decisions and Choices about Fertility and Family Planning: Perspectives from Husbands and Wives in Sudan.

Thank you for availing me the opportunity to re-submit the revised manuscript.

As my ongoing PhD thesis, this manuscript is part of my larger study, which examines interrelated aspects of fertility and family planning.

This study was conceptualised with the understanding that in Sudan, married women’s low access to family planning has a detrimental impact on their reproductive health or results in unplanned pregnancies.

In previous studies in Sudan, the low contraceptive use of married women was associated with factors concerning their low socio-economic status, misconceptions of the use of contraceptives, and other cultural practices leading to problems in accessing and using family planning services.

While these studies provide valuable evidence about family planning use, The researcher's position and previous professional experience in reproductive health have allowed her to recognize that discussions about women alone, like discussions about men separately, is an insufficient approach to understanding fertility and family planning practices. In Sudan, men, in general, are notably understudied in fertility deliberations and the natural realm of family planning literature. Their influence and role as supportive or unsupportive to their wives in the Sudanese context needs to be examined.

Furthermore, in Sub Saharan African context and elsewhere, particularly where fertility remains high, a sizeable mass of studies has claimed the importance of scaling up family planning programs to examine gender norms whilst recognizing men’s role in their partner's contraceptive use. However, what is missing from the literature is to what extent are men’s behaviours and attitudes influencing a women’s fertility and family planning use.

This study, therefore, aims to present a more nuanced understanding of whether husbands' fertility preferences are actually reflected in their wives' family planning practices and how and when their wives adopt their wishes in their actual family planning use.

As mentioned in my previous letter dated March 2024, given the current ongoing war in Sudan, we hope that in the not-so-far future, Sudan will get back on track and the significance of this paper will contribute to realising underlying factors that relate to the country’s low contraceptive prevalence rate.

In this regard, I would like to put forth the following comments.

**What is known about this topic**

- Ample scientific evidence from sub-Saharan Africa and elsewhere has reported the role of men’s decision-making role in family planning.
- Men’s disapproval and lack of engagement in family planning contribute to women’s low levels of contraceptive use.
- There are cultural traditions perceived to influence women’s use of family planning in contexts with high fertility.

**What this study adds**

- This is one of few studies in a sub-Saharan African setting and the first in Sudan to match the husband and wife's responses in fertility and family planning literature.
- The study provides information about the socio-cultural influences that shape divergent perceptions of fertility preferences and family planning use of women.
- The study presents findings based on the context and extent to which wives are willing to concede or overrule men’s preferences in fertility and family planning.

Response to Academic editor, reviewers 1 and 2 are noted below. Please note that there are episodes of overlap in responses to the reviewers, but that is because there are some similarities in points put forward by them.

**Academic Editor:**

| When submitting your revision, we need you to address these additional requirements. | Please see below. |
| --- | --- |
| We note that this data set consists of interview transcripts. Can you please confirm that all participants gave consent for interview transcript to be published?  If they DID provide consent for these transcripts to be published, please also confirm that the transcripts do not contain any potentially identifying information (or let us know if the participants consented to having their personal details published and made publicly available). We consider the following details to be identifying information: - Names, nicknames, and initials - Age more specific than round numbers - GPS coordinates, physical addresses, IP addresses, email addresses - Information in small sample sizes (e.g. 40 students from X class in X year at X university) - Specific dates (e.g. visit dates, interview dates) - ID numbers | In The methods section of the study it has been mentioned that the participants have been informed of the study's nature and purpose. Accordingly, all eligible participants agreed to participate and were assured of anonymity. Confirming this point, as shown in the results section of the study, quotations from the study participants were used to reflect and respond to the study’s main aim. captions/quotes. The names, specific ages, addresses, etc., are not shown in the study. |
| Please amend either the title on the online submission form (via Edit Submission) or the title in the manuscript so that they are identical. | The title has been amended as per comments from reviewer 2. |
| Please include captions for your Supporting Information files at the end of your manuscript, and update any in-text citations to match accordingly. | As mentioned in the methods section, Annex 1 shows questions that guided the interviews with participants. Tables 1 and 2, included in the study, show the socio-demographic background of the participants and the use of the couple’s family planning methods, respectively**.** In-text citations/quotes do match according to socio-demographic characteristics, as shown in Table 1**.** |
| If you would like to make changes to your financial disclosure, please include your updated statement in your cover letter. Guidelines for resubmitting your figure files are available below the reviewer comments at the end of this letter. | No changes |
| We look forward to receiving your revised manuscript. | I look forward to a fruitful collaboration and a beneficial contribution to the family planning literature about Sudan. |

**Reviewer 1:**

| **Comment:**  Lack of Novelty:  The study contributes little new knowledge to the existing literature. The influence of male dominance in family planning decisions, particularly in patriarchal societies, is well-documented in various studies across sub-Saharan Africa. This manuscript does not offer new insights or significant theoretical advancements in understanding these dynamics. | As some studies indeed show men’s decision-making role in women’s family planning, The authors of this study have critically reviewed it to contribute to the literature of family planning and reproductive health to address specifically to what extent are men’s fertility perceptions and attitudes actually influencing and reflected in their wives' contraceptive use and by explaining how their wives' react to their husbands' influences. |
| --- | --- |
| Specific Comments:  Introduction:  The introduction provides a broad overview of the issue but lacks a clear statement of the research question. It would benefit from a more focused articulation of the study’s objectives and how it aims to fill specific gaps in the existing literature. | The introduction has been revised to posit Sudan’s high fertility contexts and the low contraceptive prevalence rate and what factors might contribute to this. The study also highlights the men’s fertility desires as one of the reasons behind a women’s low contraceptive use, which is relevant to the context of Sudan. The introduction ends with the main aim of the study, as indicated above. Moreover, studies from Sub-Saharan Africa and elsewhere do present men’s decision-making behaviors; however, to what extent these behaviors influence women are still under studied. Some agree to their decisions, yet other women may not for several reasons, which may indicate a shift in power dynamics among married couples. Furthermore, the examination of responses from husbands and their wives presents a more nuanced understanding in this regard. |
| Methodological Flaws:  The qualitative methodology employed is insufficiently rigorous. The study’s sample size of 46 participants (23 couples) is small and lacks diversity, limiting the generalizability of the findings. Additionally, the recruitment process using a snowball sampling method introduces significant bias, as participants are likely to share similar sociocultural backgrounds, further reducing the representativeness of the sample. The data collection and analysis methods are described in vague terms, with insufficient detail about how thematic coding was conducted. The use of NVivo software is mentioned, but without a clear explanation of the coding process, it is difficult to assess the reliability and validity of the results. | Regarding the scope of this study's participants, inclusion was continued until saturation was observed, and accordingly, 46 participants (23 Couples) representing different socio-economic, religious, and educational backgrounds and family sizes were included.  The methodology has been revised, and a detailed description using subheadings identifies the procedure and validity of the study results. |
| Research Methods:  The description of the qualitative approach is inadequate. The manuscript should provide more detail on the interview process, including how questions were structured and how interviews were conducted. The justification for the chosen sample size and the use of snowball sampling is weak, and these limitations should be acknowledged more transparently. | More detail about interviewing, selection of participants purposively from the catchment areas of the family planning centers in Khartoum and Omdurman areas and how interviews with the married couple has been detailed in the methods section.  In the limitation section, it is mentioned that a larger sample for future research in a similar study is recommended. However, using a qualitative approach allows for more in-depth examination of perceptions and behaviours responding to questions of how and when husbands and wives perceive the use of family planning. |
| Results:  The results section is overly descriptive and lacks critical analysis. The themes identified from the interviews are not sufficiently supported by direct quotes or examples from the data, making it difficult to assess the validity of the interpretations. | The results section has been revised and amended based on data collected from the participants. The use of quotes in the study allows the reader to engage more closely with the perception and behavior of the participants. For example, in the interviews, some of the husbands referred to local proverbs, which are an explanation of how men in the Sudanese context view and justify their positionality when it comes to making decisions. this provides an opportunity for the reader to learn about the underlying reasoning for masculine behaviors. However, this does not engulf/encompass the whole of the study, recognizing comments of reviewers point about heavily emphasising patriarchs. It only lays the ground to contextualize how wives, despite recognizing patriarchs, may overrule such dominance. |
| Overemphasis on Patriarchy without Critical Analysis:  The manuscript heavily emphasizes the role of patriarchy in shaping family planning decisions but fails to critically analyze this context. The discussion lacks depth and does not engage with alternative explanations or theoretical frameworks that could provide a more nuanced understanding of the findings. For instance, the role of economic factors, education levels, or healthcare access is not adequately explored. | The majority of the husbands shared the concept of patriarch in this study regardless of their economic and educational characteristics, as indicated in the results section, largely based on their socialised upbringing. However, a wife’s response to these notions of masculinity may not be applicable to all of them, particularly in view of their reproductive health and contractive needs. |
| Discussion:  The discussion fails to engage critically with the findings. It merely restates the results without offering deeper insights or considering alternative interpretations. The manuscript would benefit from a more critical engagement with the literature and a discussion of the broader implications of the findings. | The discussion has been reviewed and revised to engage with the findings, |
| Repetitiveness and Redundancy:  The manuscript is repetitive, particularly in the results and discussion sections. The same points about male dominance and decision-making power are reiterated multiple times without adding substantial new information or analysis. This repetition detracts from the manuscript's overall clarity and coherence. | The results have been revised to highlight the emerging themes, which cover specifically the study's argument: agreement over fertility and family planning, disagreement about fertility and family planning, and the change of power deliberated by the wives in addressing their contraceptive needs in spite of being aware of notions of husbands' influence. They have done so in different ways to safeguard their reproductive health**.** |
| Limited Practical Implications:  While the study aims to inform policymakers about the importance of male involvement in family planning, it does not provide actionable recommendations or innovative strategies to address the identified issues. The suggestions made are generic and lack specificity, reducing the potential impact of the research on policy and practice. | The conclusion has been revised to posit the future role of family planning providers and relevant stakeholders to recognize that gender approach could be integrated into future family planning programs is essential for its success and that a women’s empowerment and visibility to attain her contraceptive choice can be achieved then. Also, men’s family planning literacy is an important step forward to recognise the importance of family planning use and its health benefits for women. |
| Ethical Considerations and Reflexivity:  The manuscript does not adequately address ethical considerations or the potential impact of the research on participants. Given the sensitive nature of the topic, more attention should have been given to ensuring confidentiality, especially considering the patriarchal context where discussing family planning can have serious social implications. Additionally, there is a lack of reflexivity regarding the researchers' positions and how this might have influenced the study. | Given the sensitive nature of this study with particular reference to husbands, participants were informed about study objectives, and after obtaining their verbal consent and agreeing to participate, the interviews were conducted by male and female data collectors, each corresponding to the same gender. Interviews were also conducted at the discretion of the participants in terms of time and place. However, always in separate spaces so that responses are not heard or influenced by either of the couple. |
| Conclusion:  The conclusion does not effectively summarize the contributions of the study or suggest clear directions for future research. It should offer more specific recommendations for policy and practice, grounded in the study’s findings. | This has been amended to summarise what the study recommends for future family planning programs for the Sudan context, which can also be relevant to other similar contexts of high fertility women’s low contraceptive use and lack of men's involvement. |

**Reviewer 2:**

| **Comment:**  TITTLE   - Improve the title by unnecessary word. - Title to be reversed | the manuscript title and keywords have been changed |
| --- | --- |
| ABSTRACT | This section has been revised to reflect a summary of the amended study**.** |
| INTRODUCTION   - NOT well addressed with your title be specific. I comment to be reversed. - Improve on writing much citation little voice of those researchers - Should explained the role of husband and how will influence family planning - You explain little modern family planning but natural family planning not well explain - And how about 9% is prevalence of modern or natural be specific and improve | The introduction has been revised to explain the contexts of fertility and family planning use in Sudan. The role of husbands had been highlighted but not in depth so as to avoid redundancy in the study as this has been amassed by different studies from the region. The contraceptive prevalence rate of both modern and natural contractive methods among married women has been included in the study. |
| METHODS   - How do you perform the interview? - What are the roles of researcher data collector? - Use the researcher scientific words - It’s thematic or content analysis makes it clear - What is the procedure done? - How trustworthiness was ensured? - Ethical consideration should be well described to PLOS ONE guideline? | The interviewing process was done as follows:  The medical staff of the two centers assisted in identifying the study participants, who were drawn purposively from both the centers' catchment areas.  Both centers are centrally located and provide extensive reproductive health services to the local community.  a team of four female data collectors interviewed the wives, and five male data collectors interviewed the husbands. Male participants are more comfortable with same-gender data collectors especially that For many Sudanese men, discussing openly any marital matters and family planning use are sensitive topics and usually considered private.  the study paid close attention to factors that may shape or influence spouses' responses. To account for this, individual interviews with the husbands and the wives were conducted on the same day with the same start and finish time but always in separate spaces.  fifteen couples were interviewed at their homes, six in their workplace, and two couples at AFHC and FRHC respectively.  regular meetings were convened between the main researcher, data collectors, and medical staff at AFHC and FRHC, to review work progress. and to validate the data generated by research questions  Ethical approval was obtained from the ethical review and research board of Ahfad University for Women (AUW), in Omdurman.  Local permission for the fieldwork was obtained from the two participating family planning centers: AFHC in Omdurman and FRHC in Khartoum.  Verbal consent for the interviews was obtained from all the study participants. |
| - What is your study design? I comment mention the study design then approach - You interview the husband or their wives make it clear | The methods section provides a clearer description of the procedure for interviewing the couple.  Both husband and their wives were interviewed. |
| RESULT   - On part of result I comment to include the social demographic table. - Also I comment using term majority on your result. - I comment writing the number of respondent when you make the quotation. - Also the tittle does not relate with the result especially in the quotations there is no husband role decision making explained its seem like you explain more perceptions and factors of decision making due to their wives. Make it reverse or change your title to write perceptions - In the part of questions are too long and appeared to be like sentences. Improve the quotations | social demographic tables have been included in the results section.  Term majority in analysis of the results has been used.  A small description of the respondent after each quote in the results section has been written, which is in accordance with Table 1.  Titles in the results sections have been changed which are more reflected in the quotes.  Quotes have been improved and are clearer. |
| REFFERENCE   - Should be reversed are not clear in PLOS ONE guideline | References have been amended following the PLOS referencing format |
| DISCUSSION   - Should be reversed. - I comment relate with the result | The discussion section has been redone to be more focused and reflect the results and main study objective. The main study will also focus on how husbands' perspectives impact their wives' actual family planning use and whether and how the wives react to their husbands' decisions. |

Inspired by your esteemed journal about the practical implications of cultural and social aspects in reproductive health-related issues I wish to re-submit this study in PLoS ONE.

We have no conflicts of interest to disclose.

Please address all correspondence concerning this manuscript to me at badrid21@gmail.com.

Sincerely

Dina Badri
