# Supplementary material for: Decisions and choices about fertility and family planning: Perspectives from husbands and wives in Sudan
Source: PLoS One. 2026 Mar 13;21(3):e0343941. doi: 10.1371/journal.pone.0343941 (PMC12987423; doi:10.1371/journal.pone.0343941)
Supplement: Attachment — Submitted filename: Response_to_Reviewers_auresp_2.docx [file pone.0343941.s003.docx]

Corresponding author: Dina Badri,
Ahfad University for Women, Sudan.

email: badrid21@gmail.com

Co-author: Professor Anja Krumeich, Dept. of Health, Ethics and Society, Maastricht University, CAPHRI,

Universiteitssingel 40, 6229 ER, Maastricht,

The Netherlands.

Email: [a.krumeich@maastrichtuniversity.nl](mailto:a.krumeich@maastrichtuniversity.nl)

Co-author: Professor Bart Van de Borne, Dept. Of Health Promotion, Maastricht University, CAPHRI, Universiteitssingel 40, 6229 ER, Maastricht, The Netherlands.

Email: [b.vdborne@maastrichtuniversity.nl](mailto:b.vdborne@maastrichtuniversity.nl)

Date: 4^th^ March 2025

To the Academic Editor PLoS One

Subject: Re-submitting the revised manuscript. Title: Decisions and Choices about Fertility and Family Planning: Perspectives from Husbands and Wives in Sudan.

Inspired by your esteemed journal about the practical implications of cultural and social aspects in reproductive health-related issues, thank you for allowing me to re-submit the revised manuscript.

As mentioned before in previous letters as part of my ongoing PhD thesis, this manuscript is part of my larger study, which examines interrelated aspects of fertility and family planning in Sudan.

Dear Academic editor, with a heavy heart, I would also like to share with you the sad news of the loss of Professor Bart Van de Borne, one of the co-authors of this study from Maastricht University. The professor passed away in February 2025. The professor’s contribution throughout this manuscript was significant. May Allah rest his soul.

Please find a response to the Academic editor, reviewers 2 and 3 noted below.

**Academic Editor:**

| Please review your reference list. Changes to the reference list should be mentioned in the rebuttal letter | Revisions have been made accordingly within the text and the reference list. The additions and omitted references shown below are better suited to the revised manuscript.  The added reference are:  Report of the International Conference on Population and Development Cairo, 5-13 September 1994. UN 1995.  Bogale B†, Wondafrash M†, Tilahun T†, Girma E. Married women’s decision making power on modern contraceptive use in urban and rural southern Ethiopia. BMC Public Health. 2011;11(342):1–7.  Center for Reproductive Rights, United Nations Population Fund. The right to contraceptive information and services for women and adolescents 2010. Available: https://www.unfpa.org/sites/default/ files/resource-pdf/Contraception.pdf  The omitted references are:  Sunmola AK, Olaosebikan JS , Adeusi TJ. High fertility level in Sub-Saharan Africa: implication for reaping and optimizing demographic dividend. International Journal of Community Medicine and Public Health. 2020; 7(9):3698-3704 <http://www.ijcmph.com>  Bongaarts J. Trends in fertility and fertility preferences in sub-Saharan Africa: the roles of education and family planning programs. Genus 76. 2020; https://doi.org/10.1186/s41118-020- 00098-z.  Upadhyay UD, Karasek D. 2010 Women’s empowerment and achievement of desired fertility in Sub-Saharan Africa. DHS Working Papers 80. 2010; Calverton, MD: ICF Macro.  Gele, AA , Shrestha M , Sheikh NS , Qureshi SA. Pregnant and powerless: exploring barriers to contraceptive use among women in Mogadishu, Somalia. Health Services Research and Managerial Epidemiology. 2022; 9:1-7. DOI: 10.1177/23333928221117057. 21  Baiden F, Mensah GP, Akoto NO, Delvaux T, Appiah PC. Covert contraceptive use among women attending a reproductive health clinic in a municipality in Ghana. BMC Womens Health. 2016;16(1)1–10.  Anbesu EW, Aychiluhm SB, Alemayehu M. Women’s decisions regarding family planning use and its determinants in Ethiopia: A systematic review and meta-analysis protocol. PLoS ONE. 2022; 17(10): e0276128. https://doi.org/ 10.1371/journal.pone.0276128  MWSS (Ministry of Welfare and Social Security) & UNDP. 2012. Sudan National Human Development Report. UNDP, Khartoum.  El Nagar S. Women and work in Sudan, problems, challenges and prospects. 1996. Paper presented at CAWTER, Tunisia. |
| --- | --- |
| Financial disclosure | No changes |

**Reviewer 2:**

| Add a table showing the social demographics of your respondents. | Table has been included, please refer to the results section. |
| --- | --- |
| Indicate the study design and approach clearly | Methodology has been revised to clarify design and approach. |
| How did you ensure trustworthiness in your study | Methodology has been revised to clarify the trustworthiness of study |
| Indicate clearly whether you used thematic or content analysis | Methodology has been revised to clarify this |
| Kindly revise and improve upon your limitations. | Limitation revised |
| Kindly make sure to proofread your work and correct all grammatical and typographical errors | Re-read and corrected. |

**Reviewer 3:**

| Introduction: This section needs revision. There are multiple short paragraphs, they lack continuity and do not establish facts in a coherent manner. | The introduction has been changed and structured to reflect continuity and coherence. |
| --- | --- |
| The quantitative information about respondents should be given as summary tables and not for each respondent | S1 table presenting socio-demographic details and S2 table presenting the number of women using family planning has been summarised. |
| The qualitative results need further efforts by the authors. They are given in a very superfluous manner and lack an in-depth analysis. The themes and sub-themes emerging from the data need to be described in more detail. You explain little modern family planning but natural family planning not well explained. | The results section has been re-written.  The use of participants’ narratives and the emerging analysis from the data presents a clearer results section. |
| I cannot comment on this section as the Results are not described comprehensively | The discussion section has been re-written to present a summary of findings in a concise manner. |

We have no conflicts of interest to disclose.

There have been no changes to financial disclosure.

Please address all correspondence concerning this manuscript to me at badrid21@gmail.com.

Sincerely

Dina Badri
