# Supplementary material for: Decisions and choices about fertility and family planning: Perspectives from husbands and wives in Sudan
Source: PLoS One. 2026 Mar 13;21(3):e0343941. doi: 10.1371/journal.pone.0343941 (PMC12987423; doi:10.1371/journal.pone.0343941)
Supplement: Attachment — Submitted filename: Response_to_reviewers_auresp_3.docx [file pone.0343941.s004.docx]

Corresponding author: Dina Badri,
Ahfad University for Women, Sudan.

email: badrid21@gmail.com

Co-author: Professor Anja Krumeich, Dept. of Health, Ethics and Society, Maastricht University, CAPHRI,

Universiteitssingel 40, 6229 ER, Maastricht,

The Netherlands.

Email: [a.krumeich@maastrichtuniversity.nl](mailto:a.krumeich@maastrichtuniversity.nl)

Co-author: Professor Bart Van de Borne†, Dept. Of Health Promotion, Maastricht University, CAPHRI, Universiteitssingel 40, 6229 ER, Maastricht, The Netherlands.

Email: [b.vdborne@maastrichtuniversity.nl](mailto:b.vdborne@maastrichtuniversity.nl)

Date: 10^th^ May 2025

To the Academic Editor PLoS One

Subject: Re-submitting the revised manuscript. Title: Decisions and Choices about Fertility and Family Planning: Perspectives from Husbands and Wives in Sudan.

Thank you for allowing me to resubmit the revised manuscript, inspired by your esteemed journal's article on the practical implications of cultural and social aspects in reproductive health-related issues.

As mentioned in previous letters as part of my ongoing PhD thesis, this manuscript is part of my larger study, which examines interrelated aspects of fertility and family planning in Sudan.

Please find a response to the Academic editor, reviewers 2 and 3 noted below.

**Academic Editor:**

| Please review your reference list. Changes to the reference list should be mentioned in the rebuttal letter | The text and the reference list have been revised accordingly. The additions and omitted references below are better suited to the revised manuscript.  **Added references**:  Caldwell JC, Caldwell P. The Cultural Context of High Fertility in Sub-Saharan Africa. *Population and Development Review. 1987;* 13 (3): 409–437. doi:10.2307/1973133.  Musalia J. Extra conjugal determinants of spousal communication about family planning in Kenya. Sex Roles. 2003; 49 (11): 597-607. doi: [10.1023/B:SERS.0000003130.04774.a1](https://www.researchgate.net/deref/http%3A%2F%2Fdx.doi.org%2F10.1023%2FB%3ASERS.0000003130.04774.a1)  Upadhyay UD, Karasek D. Women’s empowerment and achievement of desired fertility in Sub-Saharan Africa. DHS Working Papers 80. 2010; Calverton, MD: ICF Macro. 2010.  Bankole A, Ezeh AC. Unmet Need for Couples: An Analytical Framework and Evaluation with DHS Data. *Population Research and Policy Review. 1999.* 18 (6): 579-605. <https://doi.org/10.1023/A:1006373106870>  Kodzi I, Johnson D. Casterline JB. Examining the Predictive Value of Fertility Preferences Among Ghanaian Women. [*Demographic Research*](https://www.researchgate.net/journal/Demographic-Research-1435-9871) 22. 2010; (30): 965-984. doi:[10.4054/DemRes.2010.22.30](http://dx.doi.org/10.4054/DemRes.2010.22.30).  Randall S, Mondain N, Diagne A. Men, Fertility Control and Contraception in Senegal. Paper presented at Sixth African Population Conference, Ouagadougou. INDEPTH network, ACCRA Ghana, December 2011.  Anbesu EW, Aychiluhm SB, Alemayehu M. Women’s decisions regarding family planning use and its determinants in Ethiopia: A systematic review and meta-analysis protocol. PLoS ONE. 2022; 17(10): e0276128. https://doi.org/ 10.1371/journal.pone.0276128  Ahmed HM. Barriers to family planning in Sudan: Results from a survey in White Nile, Kassala and Al‐Gadarif, 2008. African Development Review. 2013; 25 (4): 499–512.  Patton MQ. Qualitative Evaluation and Research Methods (2nd ed.). Newbury Park, CA: Sage. 1990.  Nyimbili F. Nyimbili L. Types of purposive sampling techniques with their examples and application in qualitative research studies. British Journal of Multidisciplinary and Advanced Studies: English Lang., Teaching, Literature, Linguistics & Communication. 2024; 5(1),90-99  Bankole, A. and Singh, S. ‘Couples’ fertility and contraceptive decision-making in developing countries: Hearing the man’s voice’. International Family Planning Perspectives. 1998*;* 24:2. DOI: 10.2307/2991915  Alnory, AH. Islam, Women and Fertility in Sudan. *Gezira Journal of Economic and Social Sciences* 2. 2010; University of Gezira, Medani – SUDAN.  Blacker J, Opiyo C, Jasseh M, Sloggett A, Ssekamatte-Ssebuliba J. Fertility in Kenya and Uganda: A comparative study of trends and determinants. Popul Stud. 2005; 59(3):355–373.  Bawah AA, Akweongo P, Simmons R, Phillips JF. Women’s fears and men’s anxieties: The impact of family planning on gender relations in northern Ghana. Studies in Family Planning. 1999; 30(1): 54–66.  Agarwal B. Bargaining and gender relations: Within and beyond the household. Feminist Economics. 1997; 3:1:1–51. [doi.org/10.1080/135457097338799](https://doi.org/10.1080/135457097338799)  Wambete SN, Serwaa D, Dzantor EK, Baru A, Poku-Agyemang E, Kukeba MW, et al. Determinants for male involvement in family planning and contraception in Nakawa Division, Kampala, Uganda; An urban slum qualitative study. PLOS Glob Public Health. 2024; 4(5): e0003207. https://doi.org/10.1371/journal. pgph.0003207  Johnbosco M, Love O, Chuma E, Christian M, Chukwunenye I. Ifeoma E. Male involvement in family planning; An often neglected determinant of contraceptive prevalence in Sub-Saharan Africa. International Journal of Scientific Reports. 2019; 260–265; DOI:https://doi.org/10.18203/issn.2454-2156.IntJSciRep20193766.  Biddlecom AE, Fapohunda BM. Covert contraceptive use: Prevalence, motivations, and consequences. Stud Fam Plan. 1998;29(4):360–72.  Kibira SPS, Karp C, Wood SN, Desta S, Galadanci H, Makumbi FE, et.al. Covert use of contraception in three sub Saharan African countries: A qualitative exploration of motivations and challenges. BMC Public Health. 2020; 20:865. https://doi.org/10.1186/s12889-020-08977-y  Adongo PB, Tapsoba P, Phillips JF, Tabong PT, Stone A, Kuffour E, et. al. The role of community-based health planning and services strategy in involving males in the provision of family planning services: A qualitative study in Southern Ghana. Reproductive Health. 2013; 10 (36): 1–15.  Kabagenyi A, Jennings L, Reid A, Nalwadda G, Ntozi J, Atuyambe L. Barriers to male involvement in contraceptive uptake and reproductive health services: a qualitative study of men and women's perceptions in two rural districts in Uganda. Reproductive Health. 2014; 11:1–9.  health clinic in a municipality in Ghana. BMC Womens Health. 2016;16(1)1–10.  **Omitted Reference**:  Roudsari RL, Farangis S, Goudarzi F. Barriers to the participation of men in reproductive health care: a systematic review and meta-synthesis. BMC Public Health. 2023; 23(818).https://doi.org/10.1186/s12889-023-15692-x  Ratcliffe AA, Hill AG, Walraven G. Separate lives, different interests: male and female reproduction in the Gambia. Bull World Health Organ. 2000;78(5):570–579.  Schuler S, Rottach E, Mukiri P. Gender norms and family planning decision-making in Tanzania: a qualitative study. Journal of Public Health in Africa. 2011;5:2:e25. DOI:10.4081/jphia.2011.e25. 19 |
| --- | --- |
| Financial disclosure | No changes |

**Reviewer 2:**

| The abstract is well-structured; however, consider summarizing the key findings in bold points for easier readability. | This had been amended, please see the abstract section.  . |
| --- | --- |
| Clearly define key terms early on, such as "family planning," "contraceptive use," and specific cultural terms related to fertility in Sudan | This has been amended; please see some changes in the introduction section. |
| The authors could strengthen the rationale for the study by explicitly stating the gaps in existing research and the significance of addressing these in the Sudanese context | This has been amended; please see the last section in the introduction. |
| The authors should have provided strong details on methodology; however, clarify the rationale behind the sample size (46 | This has been mentioned in the data collection and trustworthiness sections of the study.  “There was no set limit to the number of interviews for the study. Saturation was reached when no new data emerged. Therefore, a total of 46 participants were included in this study.” |
| The decision to conduct interviews separately | This has been mentioned in the data collection section.  “Drawing from the work modelled in Zipp and Toth [40, 41], the study paid close attention to factors that may shape or influence spouses' responses. To account for this, individual interviews were conducted on the same day with the same start and finish time, but always in separate spaces.” |
| Consider including additional information on any pilot testing done for interview guides or data collection methods to enhance credibility. | A summary of this is mentioned now in the data collections methods section. “Drawing upon literature on contraceptive decision making and the role of men and women in contraceptive decisions in Sub-Saharan Africa and other similar relevant contexts, questions were conceptualized but designed and formulated in accordance to specifically address the Sudanese context.  Annex 1 has a list of the interview guide questions for participants used in the study. |
| The four pathways are insightful. To enhance clarity, consider summarizing each pathway with a diagram or table that encapsulates the findings visually. | Table added in the results section. S2 table. |
| When quoting participants, ensure a uniform presentation style to enhance readability. • Tables (S1 and S2): Ensure that the tables are referenced consistently in the text. For instance, include contextual details before referencing a particular table. | Revised: This has been changed, please refer to the new, concise description of couples in brackets at the end of each quote, for easier readability |
| Discussion • Integrate relevant theoretical frameworks that support your findings. This could enhance the academic level and context of your analysis. • Connect the findings to broader implications beyond Sudan, discussing how culturally influential decisions in family planning are a common issue in various regions globally. | Changes made in the discussion relate to broader findings and relevant theoretical frameworks |
| Your acknowledgment of limitations is good. It could be beneficial to offer suggestions for future research, highlighting how studies could be expanded or improved in method. | Additional title: ‘study implications’ is included in the study |
| Conclusion • The conclusion consolidates findings effectively. Reinforce the call to action for integrating men into family planning discussions, as it positions your research within a solution-focused frame. | Changes made in the conclusion sections |
| Ensure that all references are formatted consistently according to the selected citation style; this includes consistency in formatting year, volume, and pages in your references list. | Reference list revised |

**Reviewer 3:**

| The summary tables for the socio-demographic are not in the standard way. | | Revised | |
| --- | --- | --- | --- |
| The description of the qualitative approach is inadequate. The summary tables for the socio-demographic are not in the standard way.  The justification for the chosen sample size and the use of snowball sampling is weak, and these limitations should be acknowledged more transparently. | This section has been included in the methods section:  ‘The medical directors and key health services providers have extensive experience providing family planning and reproductive health services for many years in their respective communities and were, therefore, suitable to assist in identifying entry points and potential study participants in the health centres' catchment areas’.  This section has been included in the methods section:  ‘Participant recruitment and inclusion criteria: Participants were selected using purposeful sampling from the communities served by the FRHC in Khartoum and the AFHC in Omdurman. Using a snowballing approach, the medical directors and key health services providers asked potential study participants to refer further participants. This snowballing approach was beneficial in finding and including relevant participants responding to the study's objective and ensuring the accumulation of information richness. *Patton MQ. Qualitative Evaluation and Research Methods. 1990. *Nyimbili F. Nyimbili L. Types of purposive sampling techniques with their examples and application in qualitative research studies. 2024. | |  |
| The results section is still descriptive and lacks critical analysis. The themes identified from the interviews are support | Revision made for a clearer analytic approach in all 4 pathways/themes | |  |
| The discussion does not critically engage with the findings. It merely restates the results without offering deeper insights or considering alternative interpretations. The manuscript would benefit from a more critical engagement with the literature and a discussion of the broader implications of the findings. | Changes made in this section relate to broader findings and relevant theoretical frameworks. | |  |

We have no conflicts of interest to disclose.

There have been no changes to financial disclosure.

Please address all correspondence concerning this manuscript to me at badrid21@gmail.com.

Sincerely

Dina Badri
