# Supplementary material for: Decisions and choices about fertility and family planning: Perspectives from husbands and wives in Sudan
Source: PLoS One. 2026 Mar 13;21(3):e0343941. doi: 10.1371/journal.pone.0343941 (PMC12987423; doi:10.1371/journal.pone.0343941)
Supplement: Attachment — Submitted filename: Response_to_reviewers_auresp_4.docx [file pone.0343941.s005.docx]

Corresponding author: Dina Badri,
Ahfad University for Women, Sudan.

email: badrid21@gmail.com

Co-author: Professor Anja Krumeich, Dept. of Health, Ethics and Society, Maastricht University, CAPHRI,

Universiteitssingel 40, 6229 ER, Maastricht,

The Netherlands.

Email: [a.krumeich@maastrichtuniversity.nl](mailto:a.krumeich@maastrichtuniversity.nl)

Co-author: Professor Bart Van de Borne†, Dept. Of Health Promotion, Maastricht University, CAPHRI, Universiteitssingel 40, 6229 ER, Maastricht, The Netherlands.

Email: [b.vdborne@maastrichtuniversity.nl](mailto:b.vdborne@maastrichtuniversity.nl)

Date: 23 January 2026

To the Academic Editor PLoS One

Subject: Re-submitting the revised manuscript. ‘Decisions and Choices about Fertility and Family Planning: Perspectives from Husbands and Wives in Sudan’.

Thank you for allowing me to resubmit the revised manuscript, inspired by your esteemed journal's article on the practical implications of cultural and social aspects in reproductive health-related issues.

Please find a response to the Academic editor, reviewers 2 and 4 noted below.

**Academic Editor:**

| Please review your reference list. Changes to the reference list should be mentioned in the rebuttal letter | The text and the reference list have been revised accordingly. The additions and omitted references below are better suited to the revised manuscript.  **Added references**:  Schuler S, Rottach E, Mukiri P. Gender norms and family planning decision-making in Tanzania: a qualitative study. Journal of Public Health in Africa. 2011;5:2:e25. DOI:10.4081/jphia.2011.e25. 19  Comerasamy H, Read B, Francis C, Cullings S, Gordon H. The acceptability and use of contraception: A prospective study of Somalian women’s attitudes. Journal of Obstetrics and Gynaecology. 2003; 23 (4): 412–415  Atkinson R, Flint J. [Accessing hidden and hard-to-reach populations: Snowball research strategies](https://www.google.com/search?q=Accessing+Hidden+and+Hard-to-Reach+Populations%3A+Snowball+Research+Strategies&rlz=1C1RLNS_enUS903US905&oq=Rowland+Atkinson+and+John+Flint+%282001%29%3A+Authors+of+the+influential+article+%22Accessing+Hidden+and+Hard-to-Reach+Populations%3A+Snowball+Research+Strategies%2C%22&gs_lcrp=EgZjaHJvbWUyBggAEEUYOTIHCAEQIRiPAjIHCAIQIRiPAtIBCDQ5NDFqMGo3qAIHsAIB8QVKfB6AwsNYifEFSnwegMLDWIk&sourceid=chrome&ie=UTF-8&mstk=AUtExfCyRX9imqW0TjtstIpBUiMet_zh3hRX1lS5iMQlIO23sZ7Mwv2OnmeEJqfH7J2JeVSRiN05Cf-lXeNwBr3shIkOCFeFFJiWp117FJkiRRdBjOFtaTYqK2TerIG9gcY3RggpRiTaciPFSBf20sAFZ9-8XRu5fygmJKjVG7yyy3s_A94imOr6hglnnbcgbh7z4yB31Fjlq3ECgO2Oou4oqfD4EIUyO-SGt_QMq7gxjRYBMg&csui=3&ved=2ahUKEwjzg8zKkoySAxVfkmoFHeAPKBgQgK4QegQIARAB). [Social Research Update](https://www.google.com/search?q=Social+Research+Update&rlz=1C1RLNS_enUS903US905&oq=Rowland+Atkinson+and+John+Flint+%282001%29%3A+Authors+of+the+influential+article+%22Accessing+Hidden+and+Hard-to-Reach+Populations%3A+Snowball+Research+Strategies%2C%22&gs_lcrp=EgZjaHJvbWUyBggAEEUYOTIHCAEQIRiPAjIHCAIQIRiPAtIBCDQ5NDFqMGo3qAIHsAIB8QVKfB6AwsNYifEFSnwegMLDWIk&sourceid=chrome&ie=UTF-8&mstk=AUtExfCyRX9imqW0TjtstIpBUiMet_zh3hRX1lS5iMQlIO23sZ7Mwv2OnmeEJqfH7J2JeVSRiN05Cf-lXeNwBr3shIkOCFeFFJiWp117FJkiRRdBjOFtaTYqK2TerIG9gcY3RggpRiTaciPFSBf20sAFZ9-8XRu5fygmJKjVG7yyy3s_A94imOr6hglnnbcgbh7z4yB31Fjlq3ECgO2Oou4oqfD4EIUyO-SGt_QMq7gxjRYBMg&csui=3&ved=2ahUKEwjzg8zKkoySAxVfkmoFHeAPKBgQgK4QegQIARAC). 2001  Liebenberg L, Jamal A, Ikeda J. Extending youth voices in a participatory thematic analysis approach. *International Journal of Qualitative Methods*. 2020; 19, 1609406920934614. <https://doi.org/10.1177/1609406920934614>  Corbin J, Strauss A. Basics of qualitative research (3rd ed.): Techniques and procedures for developing grounded theory. Sage. 2012.  Miles MB, Huberman AM, Saldanã J. Qualitative data analysis: A methods sourcebook (4th ed.) [Kindle edition]. Sage. 2020.  Akindele RA, Adebimpe WO. Encouraging male involvement in sexual and reproductive health: family planning service providers’ perspectives. Int J Reprod Contracept Obstet Gynecol. 2013;2:119–23. 32.    Kwambai TK, Dellicour S, Desai M, Ameh CA, Person B, Achieng F, et al. Perspectives of men on antenatal and delivery care service utilisation in rural western Kenya: a qualitative study. BMC Pregnancy Childbirth. 2013;13. Available from: <http://www.biomedcentral.com/1471-2393/13/134>  Shaa El deen M, Al Rasheed F. Reproductive health and causes of infertility among men and women in Sudan. Documentary Program, Balad Fe Shasha [A Country’s View]. Sudan Television Channel [S24]. 2023.  Letshwenyo-Maruatona S. Who do Batswana men prefer: male or female health providers?. American Journal of Men’s Health. 2017; 11(6) 1642–1652. DOI: 10.1177/1557988315621727  **Omitted Reference**:  Mahfouz MS. Fertility in Northern Sudan (1979-1999): levels, trends and determinants. Paper presented at the XXVI International Population Conference of the IUSSP Morocco. 2009.  Patton MQ. Qualitative Evaluation and Research Methods (2nd ed.). Newbury Park, CA: Sage. 1990.  Mkandawire P, MacPherson K, Madut K, Odwa DA, Rishworth A, Luginaah I. Men’s perceptions of women’s reproductive health in South Sudan. Health and Place 58. 2019. |
| --- | --- |

**Reviewer 2:**

| 1.While 46 participants can provide rich qualitative data, the sample size and geographic focus on Khartoum and Omdurman may limit generalizability. Including rural areas or diverse socio-economic groups could enhance the comprehensiveness | The following is amended in the Methods section: p. 6 & 7. And limitations section: p.18 |
| --- | --- |
| 2.Given the sensitive nature of family planning and gender roles, responses might be influenced by social desirability bias, especially when discussing male involvement and women’s agency. Strategies to mitigate this, such as ensuring confidentiality or triangulating data, should be explicitly discussed. | This has been amended in the methods section: p. 7 & p. 8 |
| 3. The summary indicates common themes but lacks detail on how these themes emerged or how differences among participants were handled. A more detailed explanation of the coding process and thematic development would strengthen methodological transparency. | This has been amended in the methods section. p. 8 |
| 4.Although some wives seek to assert their reproductive choices, the paper could delve deeper into the barriers they face when doing so, including cultural, social, or economic obstacles. This would provide a fuller picture of women’s agency | This has been amended in the introduction section. p. 3 |
| 5. While recommendations are made for involving men and training health providers, specifics on how these strategies might be operationalized in the Sudanese context are limited. Including examples or frameworks could improve practical utility. | This has been amended in the study implications & conclusion sections: p. 18 & 19 |

**Reviewer 4:**

| 1.In the introduction, the author(s) should address the following: a. Abbreviations should be provided in full before using them in the paper. E.g. ICPD b. Also, perhaps it will be important for the author(s) to clearly define upfront some of the key terms they used in their study to ensure readers understand their specific meanings in the context of the study. E.g. family planning, contraceptive use, and fertility preferences.. | Pt. a is amended in introduction section: p. 3  Pt. b is amended in introduction section: p. 5 | |  |
| --- | --- | --- | --- |
| 2.The section title “aims” should be merged with the introduction. | | This has been amended in introduction section: p. 4 | |
| 3. The section titled “methodology” should be “methods.” | | This has been amended in methods section: p. 5 | |
| 4.The colon placed in the subsections in the methodology section should be removed. | | This has been amended in methods section: p. 5-8 | |
| 5.In using the snowballing approach, how did the author(s) address sample bias? This is because participants can be more inclined to recommend those who have similar traits or experiences, which could distort the sample. | | This has been amended in methods section: under sub heading Participant recruitment and inclusion criteria p. 5  . | |
| 6.What was the theoretical foundation of the study? | | This has been amended in methods section: under sub heading Trustworthiness of the study. p.8 | |
| 7.The author(s) make mention of data collectors; were these people trained? Who trained them? | | This has been amended in methods section: under sub heading data collection & study procedure p. 6 | |
| 8.S1 Table should be labelled Table 1. | | This has been amended in methods section p. 8 and (supporting files table 1) | |
| 9.In Table S1 Table, the column data collection methods should be deleted | | This has been amended in results section p. 9 and (supporting files table 1) | |
| 10.In reporting the quotes made by participants, the author(s) should put them in this form “(Muslim- Wife, monogamous, three children)” rather than “(S1 Table. Couple: three children, Khartoum).” This is to help readers appreciate who is making the comments and the context in which they are being made. | | This has been amended in results section: p. 11-16 | |
| 11. The title states that the study will explore the perspective from husbands and wives, but the quotes reported as presented by the author(s) were only from the women. Can the author clarify why? Because it will be equally important to include the husbands' perspective? | | Kindly refer to results section: pathways  1-3 p. 11-13.  The quotes indicate the couple's response (husband & their wife) | |
| 12. The limitation section should be added to the discussion section | | This has been amended in discussion section: p. 17 | |
| 13.Were the questions used for the interview guide adopted from a prior study? | | This has been amended in methods section: under sub heading Interview guide p. 7 | |
| 14.With the interview guide design, were there any piloting? If yes, how was it carried out, and did it inform the final design of the interview guide? | | a. Kindly be informed that this study did not have a pilot study prior to commencement. However, as noted in the manuscript (methods section), several steps were taken to facilitate data collection and ensure methodological rigour.  Please refer to methods section: sub heading Data collection and study procedure p. 7 for details of facilitation of data collection. for e.g. same gender data collectors for participants, conducting interviews with couples in separate spaces etc.  b. Further, the convening of frequent meetings, reviewing work progress, discussing preliminary findings, and discussing any emerging themes were important to validate data. Please  prefer to methods section subheading Data collection and study procedure p. 6  Points a & b are summarised in methods section: sub heading Trustworthiness of the study p. 8 | |

We have no conflicts of interest to disclose.

There have been no changes to financial disclosure.

Please address all correspondence concerning this manuscript to me at badrid21@gmail.com.

Sincerely

Dina Badri
